# Testing delivery of components of cognitive behavioral therapy for insomnia to breast cancer survivors by smart speaker: a study protocol

**DOI:** 10.1186/s12911-022-01902-w

**Published:** 2022-06-21

**Authors:** Claire M. Starling, Daniel Greenberg, Eric Zhou, Daniel Lewin, Allison S. Morrow, Daniel Lieberman, Callen Shaw, Hannah Arem

**Affiliations:** 1 Healthcare Delivery Research, MedStar Health Research Institute, Washington, DC USA; 2 Media Rez, Washington, DC USA; 3 Dana-Farber Cancer Institute, Boston, MA USA; 4 Sleep Health and Wellness Center, Santa Barbara, CA USA; 5 Department of Epidemiology, Milken Institute School of Public Health, George Washington University, Washington, DC USA; 6 Medical Faculty Associates, George Washington University, Washington, DC USA; 7 Department of Oncology, Georgetown University, Washington, DC USA

**Keywords:** Breast cancer, Cancer survivorship, Insomnia, Cognitive behavioral therapy, Voice-activated speaker

## Abstract

**Background:**

Insomnia is common in breast cancer survivors (BCS), affecting an estimated 30–50% of the 3.8 million BCS in the US. Insomnia is associated with health consequences for cardiometabolic and immune systems, neurobehavioral function, depression, fatigue, and quality of life and may put BCS at particular risk. While pharmacotherapy for insomnia may address symptoms in the short-term, cognitive behavioral therapy for insomnia (CBT-I) is considered the gold standard insomnia treatment. We describe our protocol to determine the efficacy of voice-activated delivery of CBT-I components on insomnia symptoms compared to a sleep education control among BCS.

**Methods:**

We will conduct a 6-week, randomized controlled trial with two arms. Intervention arm participants will receive a smart speaker device and will be asked to engage with the program daily, using a voice-activated speaker with an accompanying smart-phone app. Control participants will have access to a website with basic information about CBT-I, sleep, and breast cancer survivorship and will be asked to engage with the website as desired.

**Discussion:**

Our primary outcome is the Insomnia Severity Index total score. Secondary outcomes include sleep diary outcomes (sleep efficiency, wake after sleep onset, sleep onset latency, total sleep time, and sleep quality). This study will provide evidence on a promising modality to deliver elements of CBT-I for BCS experiencing insomnia.

*Trial Registration* ClinicalTrials.gov NCT05233800 Released 3/25/2022.

## Background

Breast cancer survivors (BCS) numbered 3.8 million in the United States as of 2019 [1]; a number that is growing due to advances in early detection and treatment. However, long-term side effects such as fatigue, insomnia, neuropathy, psychological distress, and cognitive dysfunction may impact quality of life. One such complaint, insomnia, is experienced by approximately 30–50% of BCS [2]. Insomnia can have detrimental impacts on cardiometabolic and immune system health, neurobehavioral function, depression, fatigue, and quality of life, each of which in turn affects mortality [3–5]. Causes of insomnia are multifaceted, including cancer-related physiological processes, iatrogenic effects of oncotherapies, menopause, and comorbid mood disorders associated with cancer diagnosis and psychosocial and economic stressors [6, 7]. The National Comprehensive Cancer Network and the American College of Physicians recommend cognitive behavioral therapy for insomnia (CBT-I) as the preferred treatment for insomnia [8–10]. However, CBT-I trained practitioners are scarce (even in accredited sleep centers) and a standard regimen of multiple follow-up visits can inhibit completion, particularly among cancer survivors who have a myriad health and financial concerns from cancer treatments [11]. In-person CBT-I trials in cancer centers have dropout rates of 20–40% despite considerable effort from research staff to engage patients; it is likely that in real-world settings non-adherence would be even higher and may be even worse during the current pandemic [12, 13]. To address this, automated CBT-I delivered via the internet such as SHUTi and Sleepio have been developed and shown to be effective [14–18]. While these programs provide greater accessibility for some participants [16–19], there is potential for alternative delivery methods to better reach and engage more participants.

Smart speaker devices have become commonplace in the home and use voice activation to respond to questions, record information, and carry out requests. Recent research has focused on using voice-activated devices to deliver physical activity recommendations, diet modification, perinatal health, and diabetes management content as well as supportive care for women undergoing chemotherapy in the home [20–24]. Results from our formative study suggest that use of voice-activated, smart speaker technology is appealing to BCS with insomnia, may allow for wide reach, appeal to the target population, and may more closely mimic a therapist than web-based programs [25]. Using smart speaker delivered components of CBT-I with automated response algorithms may improve adherence by providing real-time patient-tailored strategies. This protocol describes our upcoming study to test efficacy of our smart speaker delivered program to BCS with insomnia.

## Preliminary data

In Phase I of this project (R43CA232905) the feasibility and acceptability of the prototype for delivering CBT-I components using a smart speaker were assessed. During this phase, formative focus groups were first conducted and a prototype was subsequently created. Participants largely reported an interest in and perceived feasibility of using the voice-activated smart speaker to record their sleep patterns. They also largely thought that it would be feasible to address the challenges of lifestyle and sleep restriction, and expressed an interest in including relaxation in the program. Those who tested a prototype also provided formative feedback that informs our current work [25].

## Methods

### Overview

The overarching objective of this study is to determine the impact of voice-activated smart speaker CBT-I components on insomnia symptoms among BCS. We will use a randomized controlled trial to test the impact of a 6-week in-home utilization of the CBT-I smart speaker program compared to static internet-based educational content. It is hypothesized that, compared to participants who receive web-based content, participants who receive the voice-activated program will have improved Insomnia Severity Index (ISI) scores at the end of the 6-week intervention.

### Eligibility criteria

#### Inclusion criteria

1. Self-reported or documented diagnosis of breast cancer stage I–III or stage IV ECOG 0–1
2. Completed curative treatment (surgery, radiation, chemotherapy) > 3 months prior to enrollment [ongoing adjuvant therapy permitted]
3. Willingness to maintain a consistent dosing pattern if currently taking sleep medications or using cannabis for sleep
4. Females; Age 18 + 
5. Has not undergone other behavioral sleep treatment within the prior 12 months
6. A score greater than or equal to 8 on the Insomnia Severity Index
7. Able to understand and speak English

#### Exclusion criteria

1. Untreated obstructive sleep apnea disorder, narcolepsy disorder, restless leg syndrome, periodic limb movement disorder, delayed sleep phase syndrome, central apnea
2. Bipolar disorder, schizophrenia, initiation of psychological treatment within three months (Moderate ADHD, depression, and anxiety will not be exclusion criteria)
3. Alcohol or drug abuse in the prior year (Alcohol > 2 drinks/day on average or consuming 5 + drinks in a single day in the prior month)
4. Shift-work (any work schedule that falls outside of the hours of 7 am and 7 pm) in the prior three months or anticipated during the study time
5. Planned regular travel out of time zone (> 1 h) during the study period
6. Currently or planning to become pregnant during the study period

### Participant recruitment

Participants will be recruited from multiple sources. Clinic-based recruitment will be primarily conducted at two cancer institutes: the Medstar Washington Hospital Center Cancer Institute and the Dana-Farber/Harvard Cancer Center (DF/HCC). The Medstar Washington Hospital Center Cancer Institute research coordinator will screen daily patient appointment schedules for routine medical oncology visits after treatment completion to determine which patients might be eligible for the study. With permission from the treating physician, the research coordinator will call potentially eligible participants by phone or arrange to meet them in person if preferred to discuss the study, conduct screening, and complete informed consent. The DF/HCC team will use a variety of methods to find potentially eligible participants. They will use their internal system called OncDRS that queries a variety of different databases (DF/HCC cancer registry, DF/HCC Epic) to give a list of potential patients based on the pre-set criteria. The DF/HCC team will also identify women through the Young Women’s Breast Cancer Study which has enrolled 1300 young breast cancer patients and will approach those that have consented to be contacted for future research and fit the eligibility criteria, based on medical record review. Medical records will also be used to identify potentially eligible patients from the breast oncology clinics and recruit them at the time of a scheduled outpatient visit or after their visit via phone call or email. Those who answer affirmatively will be referred to MedStar Health Research Institute (MHRI) to administer the eligibility screening questionnaire.

Recruitment will also include those from the Georgetown Lombardi Comprehensive Cancer Center’s Survey, Recruitment, and Biospecimen Collection Share Resource (SRBSR), which includes nearly 2300 cancer survivors who have consented to be contacted for future research. Recruitment may expand to other MedStar Health clinical sites if enrollment is slow. Additionally, the Love Research Army of the Dr. Susan Love Foundation, a resource of 383,000 men and women interested in cancer research, will recruit participants. Local survivorship groups will also be offered brochures for distribution.

### Participant screening and randomization

Potential participants who express an interest in the study will first be given more detail about the study and screened for eligibility by phone. During the initial screening all inclusion and exclusion criteria will be assessed and self-reported sleep outcome data will be collected as measured by ISI. Questionnaires will be completed in Research Electronic Data Capture (REDCap), a 21 CFR Part 11-compliant data capture system provided by MHRI. All participants will complete informed consent prior to completing baseline forms. Participants will then complete a run-in period of 10 days, where they will be asked to complete an online sleep diary based on the Consensus Sleep Diary for at least seven days prior to randomization through the REDCap survey tool [26]. A biostatistics consultant will provide randomization assignments in a 1:1 allocation ratio using a block randomized design with random block sizes between 4 and 6. Randomization assignments for participants will be generated by the research team in the REDCap system. Subjects who consent to the study and are randomized but do not receive the study intervention may be replaced. Additionally, subjects who sign the informed consent form, are randomized and receive the study intervention, and subsequently withdraw will not be replaced.

### Intervention description

#### Smart speaker intervention

After randomization to the intervention arm participants will receive a smart speaker device with a voice-activated program and will be asked to enroll by entering baseline information via an accompanying smart phone application. Baseline information will capture sleeping patterns, sleep hygiene practice (e.g., timing and frequency of caffeine, use of electronic devices, and exercise, etc.), whether they have a bed partner, and general background information for the study. The smart phone app will be linked with the smart speaker device so that information added in any format enters the same user backend database. The MHRI research team will reach out to participants by email and/or phone after delivery confirmation to ensure that participants have set up and launched the program. Participants will also be instructed on device settings that will be pre-set for maximal security. Specifically, no identifying information will be accessible to the commercial smart speaker manufacturer or outside parties (aside from study staff) and all clinical data will be available only to the immediate research team. The research team will provide technical assistance for participants and will communicate issues to the technology developer using de-identified participant ID numbers to protect participant confidentiality.

Participants will be encouraged to complete the morning and night questionnaires daily, listen to educational content daily and listen to relaxation content or review additional written educational content on the accompanying smart phone app on an as desired basis. The morning module collects sleep diary data from the preceding night, provides most components of the CBT-I intervention, including schedule, sleep restriction, stimulus control, detailed education, and sleep hygiene training relevant to insomnia. Most recommendations and some of the education content are specifically tailored to individuals’ sleep habits based on their report on the intake questionnaire and ongoing data collection. The morning module also provides personalized recommendations for sleep schedules (including sleep restriction), collection information on daytime activities and delivers sleep education relevant to insomnia. The evening module gathers data regarding productivity, tiredness, and mood from that current day. The relaxation modules are prompted upon completion of the night module, although users can request a relaxation exercise at any time.

Prototypes for core CBT-I techniques were built and tailored to participant preferences as expressed during the focus groups in the Phase I stage [25]. These techniques have been shown to be efficacious in improving sleep outcomes in a meta-analysis of internet-delivered CBT-I and were developed to as closely as possible mimic therapist-delivered CBT-I [25]. The program was developed to deliver sleep restriction (intended to increase their homeostatic sleep drive) and stimulus control (intended to change bedtime associations from negative to positive) as primary components given their central importance to CBT-I. Other core components of the program include general sleep hygiene recommendations and psychoeducation, coaching participants on negative thoughts and how to replace them with positive perceptions of sleep. Relaxation is offered to participants starting in the first week based on early feedback that it was helpful and that participants looked forward to relaxation content. We included two- and seven-minute body scans, progressive muscle relaxation, breathing exercises, specific sensory focus, and guided imagery, which are each techniques commonly used in CBT-I.

#### Control

Participants in the control group will have access to a website with information about CBT-I, sleep, and breast cancer survivorship and will be told to engage with the website as desired. The content will be drawn from the education content that is programmed into the smart speaker device but will be edited to remove “active” components of CBT-I.

### Outcome measures

#### Primary outcome measure

Data will be collected on the ISI total score pre- and post-intervention as the primary outcome. The ISI is a seven-item questionnaire with response categories from 0 to 4 (total score 0–28) asking about sleep patterns and specifically characterizing insomnia over the 2 weeks prior. The ISI defines ‘no clinically significant’ insomnia as a score of 0–7, ‘sub-threshold’ insomnia as a score of 8–14, ‘moderate severity clinical’ insomnia’ a scores of 15–21, and ‘severe clinical’ insomnia as a score of 22–28.

#### Secondary outcome measures

Differences between participants in the CBT-I versus the control group will be derived from the self-report Consensus Sleep Diary [26]. The primary variables to be derived and compared across groups are: sleep efficiency, sleep quality, wake after sleep onset, sleep onset latency, and total sleep time.

#### Exploratory outcome measures

Among the intervention group, the System Usability Scale (SUS) and participant satisfaction with the program will be evaluated, and we will examine whether these factors affect ISI change.

#### Covariates

We will also collect information on the PROMIS 29-item scale to see how co-occurring symptoms such as fatigue and anxiety are distributed by group. Additional information to be collected includes a morning/eveningness questionnaire to assess chronotype and participant expectations and motivations. All data will be self-reported.

### Sample size

Power calculations were based on the null hypothesis of no significant mean adjusted difference between groups post-intervention on a continuous total ISI score, after controlling for baseline score using Analysis of Covariance (ANCOVA). A systematic review and meta-analysis published in 2016 showed a large effect size of d = 0.77 (95% CI 0.60–0.93) at post-treatment across seven randomized controlled trials [27]. A recent study of internet-based CBT-I among BCS found an effect size of 1.17 where the outcome was the ISI [28]. A series of required sample sizes were calculated in SAS 9.4 with covariate-outcome correlations with a desired power of 80% to detect effect sizes between 0.50 and 0.80 (medium to large). Alpha was set at 0.05 (2-sided test). Estimating a conservative effect size of 0.65 and a pre-post correlation of 0.5, 58 participants, with 29 in each arm would be required. We will aim to recruit N = 76 total (n = 38 per arm) to allow for drop out of up to 30%.

### Data collection

Study data will be entered into REDCap system provided by the MHRI. The data system includes password protection and internal quality checks, such as automatic range checks, to identify data that appear inconsistent, incomplete, or inaccurate. While participants will be recruited from multiple sites, all records will be collected and stored by MHRI and only MHRI research staff who have a need will be able to see identifiable information. Any adverse events (AEs) will be reported to the Institutional Review Board.

### Statistical analysis

The clinical trial will follow CONSORT guidelines to descriptively present means and frequencies of baseline characteristics and will follow guidelines in all analyses and reporting. *P* < 0.05 will be considered as the level of significance and will use 2-tailed tests.

Our primary outcome is based on the difference between intervention and control groups in the ISI score. We will test for assumptions of linearity of regression, homogeneity of error variances, independence of error terms, normality of error terms, and homogeneity of regression slopes. We will use analyses of covariance (ANCOVA) to compare the two groups, which controls for baseline ISI values. We will analyze the treatment effect using the intent-to-treat principle. Descriptive statistics will examine differences in baseline variables between completers and dropouts. If important differences are observed (suggesting the data are missing under a “Not Missing at Random” mechanism) or the loss to follow-up rate exceeds 10%, the primary analysis will be tested through multiple imputation of the ANCOVA model, where potential predictors of dropout will be included in the imputation model, e.g., response to a baseline question asking about the degree of motivation of the participants to stay in the study.

Additional analyses will describe mean changes within groups and test change using paired t-tests. We will also analyze whether we met the clinically relevant target for success of sub-threshold or better scores (≤ 14) among > 80% of the intervention participants. Group differences at post-intervention will be assessed by logistic regression, controlling for baseline ISI score.

We also plan to calculate differences between study arms for the following Consensus Sleep Diary metrics: sleep efficiency, wake after sleep onset, sleep onset latency, total sleep time, and sleep quality. Means and standard deviations will be calculated to examine differences between groups.

This study was reviewed and approved by the Georgetown Institutional Review Board (Study00004298). The trial is registered on clinicaltrials.gov (NCT05233800).

## Discussion

The described protocol is intended to investigate the efficacy of voice-activated, smart speaker delivered, CBT-I components on insomnia symptoms. While CBT-I has been proven to be effective in BCS with insomnia, it is not widely available to those who need it. As screening and treatment for breast cancer improves and survivors live longer, the need for feasible options for accessible insomnia treatment continues to grow.

Previous studies have used technology to offer components of CBT-I to cancer survivors, but to our knowledge none have used smart speakers or been designed to provide daily interaction and feedback. Researchers have delivered automated CBT-I to BCS (n = 255) via a web-based portal, showing improvements in sleep outcomes in a randomized controlled trial [16]. Another study among 18 BCS and 10 other cancer survivors on average 4 years after diagnosis demonstrated the efficacy of web-based CBT-I on the overall ISI as well as using sleep diary measures [13]. A larger study (n = 171) examining the initial evaluation of an internet CBT-I based intervention to improve the sleep of cancer survivors called I-Sleep was also found to be effective [17]. While these results support the use of automated, technology driven methods to deliver components of CBT-I to cancer survivors, results from Phase I of the study provides evidence to suggest the use of voice-activated smart speaker technology differs from web- or video-based technologies. Voice-activated technology allows for hands-free operation and a conversational interface, potentially increasing flexibility and efficiency.

In summary, this study will examine the effects of voice-activated technology to deliver components of CBT-I to BCS using a randomized controlled trial. The unique feature of using the smart speaker device will enable participants to interact with an internet-connected speaker, emulating the experience of an in-person provider rather than web-based programs. This study will provide evidence on a promising modality to deliver elements of CBT-I for BCS experiencing persistent sleep disruption. If we demonstrate efficacy, future applications could include tailoring to other populations with a high prevalence of insomnia.

## Data Availability

Data sharing is not applicable to this article as no datasets were generated or analyzed during the current study.

## Abbreviations

BCS: Breast cancer survivor
CBT-I: Cognitive behavioral therapy for insomnia
DF/HCC: Dana-Farber/Harvard Cancer Center
ISI: Insomnia severity index
MHRI: MedStar Health Research Institute
REDCap: Research electronic data capture
SRBSR: Survey, recruitment, and biospecimen collection share resource
